# Serological and molecular evidence of *Brucella* species in the rapidly growing pig sector in Kenya

**DOI:** 10.1186/s12917-020-02346-y

**Published:** 2020-05-11

**Authors:** James Akoko, Roger Pelle, Velma Kivali, Esther Schelling, Gabriel Shirima, Eunice M. Machuka, Coletha Mathew, Eric M. Fèvre, Victoria Kyallo, Laura C. Falzon, AbdulHamid S. Lukambagire, Jo E. B. Halliday, Bassirou Bonfoh, Rudovick Kazwala, Collins Ouma

**Affiliations:** 1 Department of Biomedical Sciences and Technology, Maseno University, Kisumu, Kenya; 2 Biosciences Eastern And Central Africa - International Livestock Research Institute, Nairobi, Kenya; 3 International Livestock Research Institute, Nairobi, Kenya; 4 Department of Public Health and Epidemiology, Swiss Tropical Institute, Basel, Switzerland; 5 Nelson Mandela African Institute of Science and Technology, Arusha, Tanzania; 6 Sokoine University of Agriculture, Morogoro, Tanzania; 7 Institute of Infection and Global Health, University of Liverpool, Liverpool, UK; 8 University of Glasgow, Glasgow, UK; 9 Centre Suisse de Recherches Scientifiques en Côte d’Ivoire, Neuchâtel, Switzerland

**Keywords:** “Pig brucellosis”, “Molecular detection”, “Molecular evidence”, *Brucella*, Serology, Kenya

## Abstract

**Background:**

Brucellosis is an emerging yet neglected zoonosis that has been reported in Kenya. Epidemiological data on brucellosis in ruminants is readily accessible; however, reports on brucellosis in pigs remain limited. This study sought to detect *Brucella* infection in pig serum by both serological and molecular techniques. Serum from 700 pigs randomly collected at a centralized abattoir in Nairobi region, Kenya were screened in parallel, using both Rose Bengal Test (RBT) and competitive Enzyme-Linked Immuno-sorbent Assay (cELISA) for antibodies against *Brucella* spp. All sera positive by RBT and 16 randomly selected negative samples were further tested using conventional PCR targeting *bcsp31* gene and real-time PCR (RT-PCR) assays targeting *IS711* and *bcsp31* genes.

**Results:**

A prevalence of 0.57% (*n* = 4/700) was estimated using RBT; none of these samples was positive on cELISA. All RBT positive sera were also positive by both PCRs, while two sero-negative samples also tested positive on RT-PCR (*n* = 6/20). *Brucella abortus* was detected in four out of the six PCR positive samples through a real-time multiplex PCR.

**Conclusion:**

The detection of antibodies against *Brucella* spp. and DNA in serum from slaughterhouse pigs confirm the presence of *Brucella* in pigs. Therefore, investigation of the epidemiology and role of pigs in the transmission of brucellosis in Kenya is needed. Further targeted studies would be useful to systematically quantify and identify the spp. of *Brucella* in pigs.

## Background

Brucellosis is a neglected zoonotic disease with a worldwide distribution [1]. It affects a broad range of wild and domesticated animals and is responsible for economic losses due to reduced milk yields, infertility and abortions in infected animals [2]. While there are numerous species of the bacterial genus *Brucella*, those most commonly associated with human infections are *B. melitensis, B. abortus* and *B. suis*, largely affecting small ruminants, cattle, and pigs, respectively [3]. Over 500,000 new human cases of brucellosis are reported annually globally [4]. Humans can get infected with brucellosis via direct or indirect contact with infected animals and through the consumption of undercooked or raw animal products. Therefore, control of brucellosis in animals can reduce new cases of human brucellosis. In Kenya, brucellosis is among the top priority zoonotic diseases for integrated *‘One Health’* control, but the focus is limited to ruminants, for which data on their importance as a significant source of human infection with *Brucella* is available [5, 6].

The species, *B. suis,* is considered as one of the three most common zoonotic pathogenic species to humans [1]. Nonetheless, data on the epidemiology of pig brucellosis in Kenya remains very sparse, with no recently generated reports. The only documented information on pig brucellosis was produced more than four decades ago, through a serological survey that reported the presence of *Brucella* antibodies in pigs with a prevalence of 0.2% [7]. Despite this, pork production and consumption are among the most rapidly growing livestock sectors in Kenya, with a predicted overall production growth rate of 203% for the period between 2000 and 2030 [8].

The Rose Bengal test (RBT) is the World Organisation for Animal Health (OIE) recommended screening test for brucellosis in animals [9]. However, several studies have reported false positivity with this test due to cross interaction with *Y. enterocolitica* O:9, which is quite prevalent in pig populations [10, 11]. The confirmation of the RBT by Enzyme-Linked Immuno-Sorbent Assays (ELISAs) also suffers from reportedly low sensitivity in pig sera [10, 11]. These limitations generally suggest that serological testing of pig serum with the recommended tests may not be ideal and that results should, therefore, be interpreted with caution.

The development of molecular-based assays for the rapid and specific detection of *Brucella* DNA has significantly advanced our understanding of host-pathogen interactions. Previous serology-based surveys traditionally assumed that *Brucella* spp. have host preference [10, 11]. Recent studies have shown that there is indeed a complex and diverse distribution of the pathogen among different hosts, further complicated by farming systems and close interactions between wildlife and livestock [12]. Quantitative, real-time PCR assays, such as those developed by Matero et al. [13] and Probert et al. [14], have also significantly increased the ease of detection of *Brucella* DNA, moreover, with the extraction of genomic material directly from clinical specimens [15]. These test options and findings have shed light on the complicated epidemiology and transmission of brucellosis between different hosts which, unfortunately, have not been applied to the pig population in sub-Saharan Africa. There is a scarcity of reported studies in Africa in detecting brucellosis in pigs by molecular techniques, besides the insufficient information on *B. suis* infection in the region. Therefore, the understanding of the role of pigs in the transmission dynamics of brucellosis remains limited. Previous studies have looked into pork value chains and their potential role in the transmission of other priority zoonoses [16, 17]. This study was therefore done to detect and identify *Brucella* spp. in pigs entering the Nairobi pork market. In so doing, we identified exciting variations to our current understanding of host species distributions and diagnostic challenges for brucellosis in pigs.

## Results

A total of 700 pigs were sampled at a central abattoir [16]. Pigs from all over Kenya were eligible for inclusion in the study; most sampled pigs originated from the central region (*n* = 427; 61%) and Nairobi (*n* = 159; 22.7%). Majority of the sampled pigs were female (*n* = 469; 67.0%). The pig sera were tested for antibodies against *Brucella* spp. using RBT and cELISA. Four out of the seven hundred sera tested by RBT were positive, while none were positive by cELISA (Table 1). The four RBT positive samples, together with 16 randomly selected RBT negative samples were also tested by PCR for detection of *Brucella* spp. DNA*.*

**Table 1:** Serological and molecular detection of *Brucella* antibodies and DNA in pig sera in Kenya

Test performed	Number tested	Number/proportion of positive
RBT	700	4 (0.57%)
cELISA	700	0 (0.00%)
Conventional PCR, genus specific target (*bcsp31)*	20	4 (20.00%)
qPCR, *IS711 and bcsp31targets*	20	6 (30.00%)
qPCR*, B. abortus* specific target (*alkB*)	6	4 (66.67%)
qPCR, *B. melitensis* specific target (*BMEI1162)*	6	0 (0.00%)

Out of the 20 samples selected for molecular analysis, 4 samples amplified the target region of interest on molecular testing using conventional PCR, giving an anticipated band of 223 base pairs. Data shown in Additional file 1: (Detection of *Brucella* DNA using conventional PCR method: Electronic supplementary material).

Six out of the 20 samples tested by real-time PCR amplified with both the *bcsp31* and the *IS711* genus-specific primers used to detect the presence of *Brucella* DNA. Amplification with the *B. abortus* primers was also achieved with 4 of these 6 samples. None of the samples amplified with the *B. melitensis* specific primers (Table 2).

**Table 2:** Results for RBT and Real-Time PCR positive samples (*n* = 6)

Samples	Source	Sex	RBT results	Conventional PCR	Genus specific (*IS711&* *bcsp31)*	*B.* *abortus* specific	*B.* *melitensis* specific
Serum	Central region Peri-urban	Female	-ve	not done	+ve	+ve	-ve
Serum	Rift valley region Peri-urban	Female	+ve	+ve	+ve	-ve	-ve
Serum	Central region Peri-urban	Female	+ve	+ve	+ve	-ve	-ve
Serum	Western region Peri-urban	Male	-ve	not done	+ve	+ve	-ve
Serum	Central region peri-urban	Male	+ve	+ve	+ve	+ve	-ve
Serum	Nairobi region Slum	Male	+ve	+ve	+ve	+ve	-ve

## Discussion

We detected the presence of *Brucella* DNA in pig sera that also screened positive using the RBT. According to our knowledge, this is the first report of molecular detection of *Brucella* spp. in pig serum in East Africa. Brucellosis is a widespread zoonotic disease and serological evidence of pig brucellosis has previously been described in Africa [15, 18–22]. However, information on *B.* spp. circulating in the pig population remains limited. South America and Southeast Asia are considered to have a higher prevalence of porcine brucellosis, as compared to the other regions, including Africa [1]. The sero-positivity of 0.57% detected in this study is consistent with findings from different serological studies conducted in other African countries such as Nigeria [19, 20, 22], Zambia [22] and Uganda [20], which reported prevalence of between 0 and 0.6%. However, our results differ with one study that reported a high sero-prevalnce of 30.6% in Benue State of Nigeria [21]. The high prevalence reported in Benue State, however, could be due to the clustering of positive animals in the targeted region, as other studies in Nigeria also reported lower prevalence rates [18, 23]. Despite the low prevalence of 0.57% detected from the pig population in this study, the risk of transmission in the pig population may grow with the rapidly growing pig production in Kenya, especially if control interventions are not put into place.

In this study, we found that four out of the six positive samples detected were of *B. abortus*, as they amplified with *B. abortus* primers [14]. The other two samples that did not amplify with the species identification primers could belong to a different species, given that the assay is designed to distinguish between *B. abortus* and *B. melitensis* [14]*.* The detection of zoonotic *Brucella* spp. in pig sera, and more so *B. abortus,* is of importance given that pigs are traditionally associated with *B. suis*, while cattle are considered to be the preferred host for *B. abortus* [1]. This finding therefore highlights the possibility for novel transmission dynamics within pigs and the need for further investigations to inform appropriate control strategies for brucellosis in Kenya and similar settings. The number of studies that have used serum samples for extraction of genomic DNA, and subsequent detection of *Brucella* DNA, has increased in the recent past [13, 15, 20]. This could be due to the fact that serum samples can be used for routine testing of brucellosis in both animals and humans since, unlike other samples such as abortion materials, swabs, hygromas and milk, they are not affected by the age, sex or physiological stage (such as pregnancy). Even though *Brucella* spp. are known to have host preference, cross-infection has previously been reported to be common in areas where mixed husbandry systems are practised [11, 24, 25].

Similarly, the presence of *B. melitensis* in pigs’ serum was recently reported in Egypt [15]. In that study, the prevalence was comparable to a previous report from Latin America where *B. melitensis* was detected in pigs [26]. In the current study, the positive pigs were sourced from peri-urban and slum areas, where small-scale farmers keep mixed herds that facilitates close contact between the different animal species. Free-range systems practised in the informal settlements, and feeding of pigs on waste from the market [27] could also contribute to the cross-transmission of *B. abortus* from cattle to pigs. The presence of *B. abortus* in pigs may not only present a zoonotic risk to non-suspecting farmers, slaughterhouse workers and pork consumers but also raises the need for further investigation on the epidemiology and pathogenicity of *B. abortus* in pigs, as well as their contribution to human infection.

The Rose Bengal Test (RBT), Buffered Plate Agglutination Test (BPAT) and ELISA are recommended serological tests for screening brucellosis in pigs [1]. However, the results from these tests should be confirmed by reference serology tests or confirmatory bacteriology and molecular techniques [10, 11, 28, 29]. Variability between the performance of different serological tests, or in the sensitivities and specificities for the same tests using pig serum in different studies, have been recorded [28, 30, 31]. This study also observed a poor agreement between the RBT and cELISA, which is similar to the findings in other studies [30, 31]. Further investigation should be carried out to evaluate the performance of these two assays on pig samples. There was a much better agreement between the conventional and qPCR. All four positive samples detected by conventional PCR (B4/B5 primers) were further confirmed by a multi-level genus- and species-specific RT-PCR. *Brucella abortus* was identified in four of the six positive samples. The agreement between molecular and serological tests raises an important consideration for these tests to be used for routine testing (surveillance) of brucellosis in livestock and wildlife.

This study had several limitations; first, not all 700 sera screened by the RBT and cELISA were tested by PCR. This could downplay the positivity proportion detected in the molecular assays used. Secondly, the time lapse between the serology (September 2018) and molecular testing (November 2019) implies that study personnel were not entirely blinded to the results of the initial screening when conducting PCR. Finally, the limited scope of the species identification technique used (*B. abortus* and *B. melitensis*) could imply that other *Brucella* spp., including a possibly novel species in this pig population, could have been missed in this study. Future studies should consider the suitability of different assays for the detection of pig brucellosis since it is an emerging area of research. All the assays used in this study are not explicitly designed for pig host testing, rather the assays are generally applied to the detection of *Brucella* antibodies and DNA. This could affect the sensitivity or specificity of detecting brucellosis in pigs, especially when a single test is used. Therefore, future studies should evaluate a range of tests for utility in testing for brucellosis within this crucial population.

## Conclusion

*Brucella* antibodies and DNA were detected in pig sera from slaughterhouses in Nairobi, Kenya. Further targeted studies to systematically quantify and speciate the strain of *Brucella* in pigs should be conducted.

## Methods

### Study site

The study was conducted at the largest pig abattoir that supplies unprocessed pork to consumers in the city of Nairobi. The abattoir is located at the outskirts of Nairobi and obtains pigs from all production regions in Kenya.

### Sampling and data collection

A total of 700 blood samples were collected from pigs in a prevalence study on pig cysticercosis, as previously described [16]. All pigs presented for slaughter from the months of October to December 2014 were eligible for sampling [16]. Based on the data available from Kenya at the time of the parent study design, an assumed prevalence of 32.8% was used, with a 95% confidence level and a precision of 5%, to obtain an adequate sample size for the estimation of the population prevalence of cysticercosis [16]. Pigs were systematically selected; the first pig presented for slaughter was sampled, followed by every fifth, to get an average of 15 pigs each day for 47 days. Approximately 10 ml of blood were collected from each pig into a plain vacutainer tube (BD Vacutainer). A brief questionnaire was administered to the pig owner (farmers or traders), to capture information including the origin/location, sex, and age of the sampled pigs. The blood samples were temporarily stored at 2–8 °C before transportation to the International Livestock Research Institute (ILRI) laboratories in Nairobi, Kenya. The samples were then centrifuged at 2500 rpm for 20 min and serum aliquoted into 2 ml sample tubes for storage at − 80 °C until testing.

### Serological testing

#### Rose Bengal test (RBT)

The Rose Bengal Test (RBT) was carried out using antigens provided by Instituto de Salud Tropical Universidad de Navarra @ Edificio CIMA AvdaPioXII, 55 E-31008 Pamplona, Spain. The testing was carried out according to the OIE protocol [9]. Briefly, all 700 serum samples and the antigen were defrosted at room temperature. About 25 μl of the sample was dispensed onto the glossy side of a white tile. An equal volume of the antigen was then dispensed beside each drop of serum. Each plate was prepared with negative and positive controls also provided with the kit. The antigen and serum were immediately mixed using a wooden splint, and the plate rocked gently for 4 minutes. Following this, the results were read immediately in a well-lit place and interpreted as either positive or negative. Samples were considered positive for RBT when there was any degree of visible agglutination at 4 minutes [9].

### Competitive enzyme-linked immunosorbent assay (cELISA) testing

The ELISA testing was done on all the 700 samples using competitive ELISA kit, COMPELISA 400 (cELISA APHA Scientific, Weybridge-UK) for detection of anti-*Brucella* antibodies. The cELISA testing was conducted as per the manufacturer’s instructions as follows; all reagents and serum samples were first brought to room temperature. Serum samples (20 μl) were added to each well of the ELISA plates that are pre-coated with purified standard sLPS antigen prepared from *B. melitensis* isolates and mixed with 100 μl of the freshly prepared conjugate. Positive and negative controls were included in each test run. After incubation at room temperature for 30 min and constant mixing on a rotary shaker, the plates were washed five times with the wash solution. Then 100 μl of chromogen substrate was added to each of the wells, incubated at room temperature for 15 min and the reaction stopped by adding 100 μl of stopping solution to each well. The Optical Density (OD) was determined using an ELISA reader (BioTek Synergy HT, BioTek Winooski, VT 05404 United States) at a wavelength of 450 nm. A plate was considered valid if the mean OD of the 6 negative controls at 450 nm was greater than 0.700, and the mean OD of the 6 positive controls was less than 0.100 [32]. The difference between the OD of the positive and negative controls had to be equal to or greater than 0.300. A cut-off was determined using the conjugate control, i.e. 60% of the mean OD of the four conjugate control wells [28, 30]. Any OD equal to or below the determined cut-off value was considered as being positive, while values above cut-off were considered negative [32].

### Molecular detection

#### Sample selection

The RBT positive samples plus additional randomly selected RBT negative samples (4 RBT negative sera selected for each of the RBT positive samples), were processed for molecular testing. We estimated the sample size based on the sample required to detect a minimum number of 4 positive events at a 95% confidence, given a positivity of 33%. This gave us a minimum sample estimate of 20. All the molecular testing was conducted at ILRI between November and December 2019.

#### Extraction and purification of DNA

Extraction of genomic DNA was done from 200 μl of the serum using QIAamp™ DNA Mini Kit, (QIAGEN, Germany), according to the manufacturer’s guidelines. Briefly, 20 μl of proteinase K and 200 μl of genomic lysis buffer were added to the source sample. The mixture was subjected to digestion, deactivation, washing and elution steps as per the manufacturer’s guidelines. The DNA quality and quantity were determined using a NanoDrop™ 2000c Spectrophotometer (ThermoFisher Scientific, USA). Stock DNA samples were stored at − 20 °C until the performance of PCR.

#### Conventional PCR detection of *Brucella* DNA

Molecular identification of the genus *Brucella* was done using two sets of primers: B4 forward (5′-TGG CTC GGT TGC CAA TAT CAA-3′) and B5 reverse (5′-CGC GCT TGC CTT TCA GGT CTG-3), as previously reported [29].

#### Real-time PCR detection of *Brucella* DNA

Real-time PCR was performed on all the extracted DNA samples using an ABI 7500 thermocycler machine (Applied Biosystems, Life Technologies, Singapore), beginning with a *Brucella* genus-level screening using two primers targeting *IS711* gene and *bcsp31* gene, respectively [13, 14]. The species identification using *B. abortus* and *B. melitensis*-specific primers and probes, was subsequently performed on DNA samples that showed any amplification with both targets *bcsp31* and *IS711* primers. This second round multiplex qPCR was performed using previously developed oligonucleotide primers and probes [14] as indicated in Table 3 below. Briefly, template DNA (4 μl) were mixed with 0.5 μM each of the primers targeting the *alkB* for *Brucella abortus, BMEI1162* for *B. melitensis* and 0.25 μM of fluorescent probe (primer and probe sequences are given in Table 3). About 10 μl of the Luna® Universal Probe qPCR mastermix (404 with UDG; New England BioLabs, MA, USA) was added to each oligonucleotide and DNA sample mixture. The reaction mixture (20 μl) was then run on an Applied Biosystems 7500 Real-Time PCR System (Applied Biosystems, USA). All the samples and control mixtures were tested in duplicate using the following parameters: 2 min of decontamination at 95 °C, followed by 10 min of denaturation and activation of polymerase at 95 °C, then 45 cycles of 95 °C for 15 s and 57 °C for 1 min. A sample was considered positive if it amplified in one or both wells with a cycle threshold (Ct) values < 39. Positive controls 16 M *B. melitensis* and 544 *B. abortus* (sourced from Friedrich-Loeffler-Institute, the *Brucella* reference lab in Germany) and non-template controls were included in all the real-time PCR runs.

**Table 3:** Primers and probes used for real-time PCR

Target	Gene targeted	Sequences of primers and probes (5′ -3′)	Fluorophore/ quencher	Reference
Genus *Brucella*	*IS711*	Forward GGCCTACCGCTGCGAAT Reverse TTGCGGACAGTCACCATAATG Probe AAGCCAACACCCGGC	FAM/−MGBNFQ	Matero, 2011 [13]
Genus *Brucella*	*Bcsp31*	Forward GCTCGGTTGCCAATATCAATGC Reverse GGGTAAAGCGTCGCCAGAAG Probe AAATCTTCCACCTTGCCCTTGCCATCA	6-FAM/BHQ1	Probert, 2004 [14]
*B. abortus*	*IS711* downstream of *alkB*	Forward GCGGCTTTTCTATCACGGTATTC Reverse CATGCGCTATGATCTGGTTACG Probe CGCTCATGCTCGCCAGACTTCAATG	JOE/BHQ1	
*B. melitensis*	*IS711* downstream of *BMEI1162*	Forward AACAAGCGGCACCCCTAAAA Reverse CATGCGCTATGATCTGGTTACG Probe CAGGAGTGTTTCGGCTCAGAATAATCCACA	Texas Red/BHQ2	

## Supplementary information

**Additional file 1. **Detection of *Brucella* DNA using conventional PCR method.

## Data Availability

All the analysed data are included in this article and its supplementary files.

## Abbreviations

cELISA: Competitive Enzyme-Linked Immuno-sorbent Assay
DNA: Deoxyribonucleic acid
ILRI: International Livestock Research Institute
OD: Optical Density
OIE: World Organisation for Animal Health
RBT: Rose Bengal Test
RT-PCR: Real Time Polymerase Chain Reaction
