# Supplementary material for: Serological and molecular evidence of Brucella species in the rapidly growing pig sector in Kenya
Source: BMC Vet Res. 2020 May 11;16:133. doi: 10.1186/s12917-020-02346-y (PMC7216537; doi:10.1186/s12917-020-02346-y)
Supplement: Supplementary file 1 — Additional file 1. Detection of Brucella DNA using conventional PCR method. [file 12917_2020_2346_MOESM1_ESM.pdf]

File 1: Detection of *Brucella* DNA using Conventional PCR

Molecular identification of the genus *Brucella* was done using two sets of primers: B4 forward (5´-TGG CTC GGT TGC CAA TAT CAA-3´) and B5 reverse (5´-CGC GCT TGC CTT TCA GGT CTG-3), as previously reported (21). The PCR reaction mix comprised of: a final concentration of 0.5µM for each of the primer pairs, 5 µl of the DNA template (4-20ng/µl) and x1 concentration of the PCR mastermix (AccuPower PCR PreMix Bioneer Corp, Republic of Korea) to a final volume of 25 µl. After the initial denaturation step of 5 minutes at 95°C in a thermo-cycler (Applied Biosystems SimpliAmp Thermo Cycler, Lite Technologies, Singapore), 35 cycles of denaturation at 94°C for 1minute, annealing at 53°C for 30seconds, extension at 72°C for 1 minute, and final extension steps at 72°C for 10 minutes were performed. Amplification of the target region was confirmed based on the presence of specific bands for *Brucella (*223 bp). The PCR products (3µl) were analysed on a 1.8% agarose gel pre-stained with GelRed nucleic acid stain (Biotium Inc., USA) run at 6.7 v/cm^2 for 30 minutes for electrophoresis detection and visualization with a bioanalytical imaging system (Azure Biosystems Inc., USA).

Results

Out of the 20 samples selected for molecular analysis, 4 samples amplified the target region of interest on molecular testing using conventional PCR, giving an anticipated band of 223 base pairs (Figure 1).

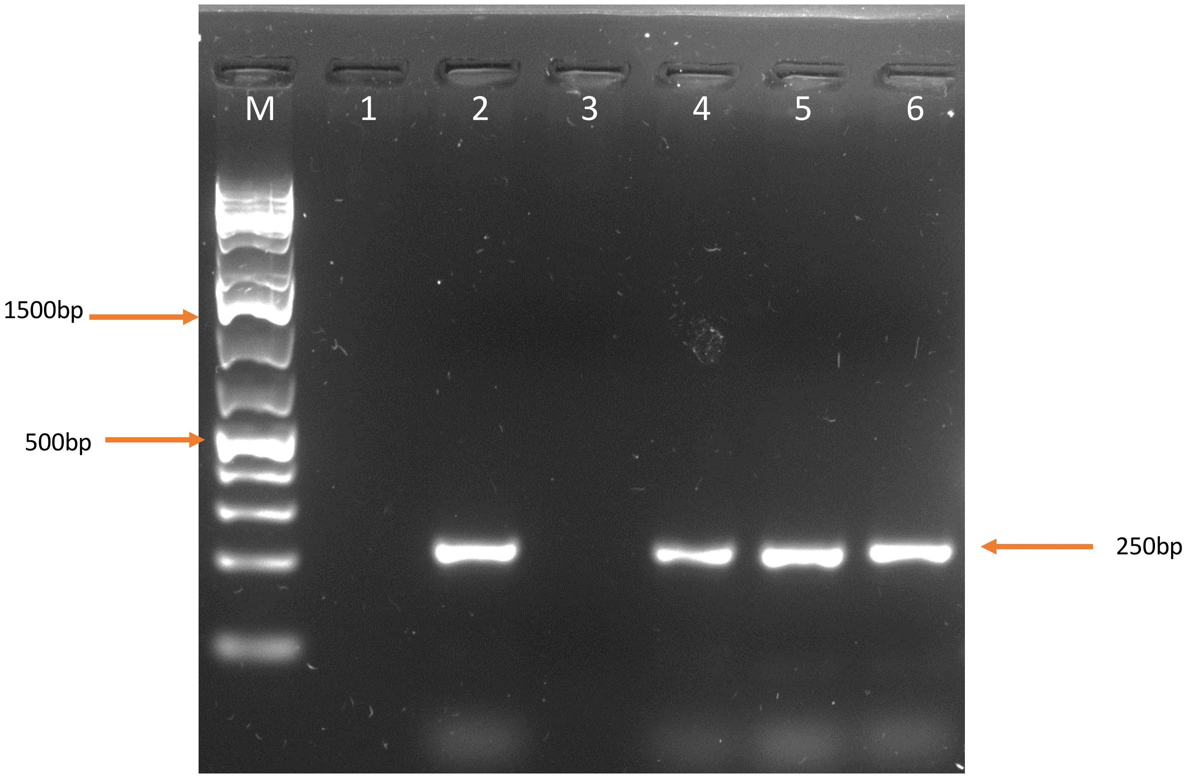

Figure 1: An agarose gel image showing a 223bp amplicon of the NTC – negative template control, samples 1-4 against a 1kbplus ladder (ThermoFisher Scientific, USA).

Six out of the 20 samples tested by real-time PCR amplified with both the bcsp31 and the *IS711* genus-specific primers used to detect the presence of *Brucella* DNA.
